# Topical use of 5% acyclovir cream for the treatment of occult and verrucous equine sarcoids: a double-blinded placebo-controlled study

**DOI:** 10.1186/s12917-017-1215-0

**Published:** 2017-10-06

**Authors:** Maarten Haspeslagh, Mireia Jordana Garcia, Lieven E. M. Vlaminck, Ann M. Martens

**Affiliations:** Department of Surgery and Anaesthesiology of Domestic Animals, Faculty of Veterinary Medicine, Ghent University, Salisburylaan 133, 9820 Merelbeke, Belgium

**Keywords:** Acyclovir, Bovine Papillomavirus, Equine Sarcoid, Topical treatment

## Abstract

**Background:**

Previous studies mention the use of topical acyclovir for the treatment of equine sarcoids. Success rates vary and since the bovine papillomavirus (BPV) lacks the presence of a kinase necessary to activate acyclovir, there is no proof of its activity against equine sarcoids.

**Results:**

Twenty-four equine sarcoids were topically treated with acyclovir cream and 25 with a placebo. Both creams were applied twice daily during 6 months. Before the start of the treatment and further on a monthly basis, photographs and swabs were obtained. On the photographs, sarcoid diameter and surface area were measured and verrucosity of the tumours was quantified using a visual analog scale (VAS). The swabs were analysed by PCR for the presence of BPV DNA and positivity rates were calculated as the number of positive swabs divided by the total number of swabs for each treatment group at each time point. Success rates were not significantly different between both treatment groups. There was also no significant effect of treatment on sarcoid diameter, surface area or VAS score. For the swabs, a significantly higher BPV positivity rate was found for acyclovir treated tumours compared to placebo treated sarcoids only after 1 month of treatment and not at other time points.

**Conclusions:**

None of the results indicate that treatment with acyclovir yields any better results compared to placebo treatment.

## Background

Acyclovir (acycloguanosine) is an antiviral drug developed for the treatment of herpes simplex virus (HSV) infections in humans [1]. The drug relies on competitive inhibition of the viral DNA polymerase, but needs to be phosphorylated by a HSV specific thymidine kinase and then further by cellular enzymes to a triphosphate form to exert its action [2]. Nevertheless, inhibition of viral replication also occurs for other viral species then HSV [3], suggesting phosphorylation of the drug may also occur in cells where the HSV thymidine kinase is not present, albeit to a lesser extent.

Equine sarcoids are tumours originating in the dermal layers of equine skin. The pathogenesis is not entirely clear yet, but there is agreement in literature that the bovine papillomavirus (BPV) (mainly type 1 and type 2) most likely plays an important role in the development of these tumours [4]. Many treatments have been reported, but no universal treatment has been found to cure all sarcoids on all body locations.

Topical treatment with acyclovir has been described to result in complete regression in 68% of occult, verrucous, nodular or mixed equine sarcoids [5]. In addition, a recent ex vivo study has shown that acyclovir concentrations reached in the dermal layers after topical administration on sarcoid-affected equine skin are high enough to possibly achieve an antiviral effect [6]. However, the thymidine kinase necessary for initial phosphorylation of the drug is missing in BPV DNA [7] and the susceptibility of the BPV DNA polymerase for acyclovir is unknown. A retrospective study further described complete regression after topical acyclovir treatment in only 53% of the cases [8].

The goal of the present study was to establish if topical treatment of occult equine sarcoids with a 5% acyclovir cream is more effective compared to the application of a placebo cream following the same administration protocol.

## Methods

### Subjects

A power analysis revealed that at least 15 unrelated sarcoids were needed in each treatment group to be able to show a difference between acyclovir and placebo treatments (power = 0.8; type-1 error = 0.05). Because multiple sarcoids on the same horse would be treated the same way, a minimum of 15 horses was necessary in each treatment group.

All horses that were presented to the Department of Surgery and Anaesthesiology of the Faculty of Veterinary Medicine of Ghent University for the treatment of previously untreated occult or partly verrucous equine sarcoids were considered for inclusion in the study. Horses that had fibroblastic or nodular sarcoids in addition to the occult and/or partly verrucous tumours and horses that received concurrent medical treatment for other indications were excluded. The diagnosis of equine sarcoid was made by clinical examination by an experienced veterinarian. As a compensation for taking part in the study, the treatments and consultations were offered free of charge and sarcoids that would have been treated with placebo during the study would be treated afterwards without additional costs. When owners were willing to participate, an informed consent was signed in which the owners also committed to apply the topical treatment as instructed.

### Treatment and sampling

Sarcoids were topically treated with either a generic 5% acyclovir cetomacrogol cream or a placebo consisting of the same cetomacrogol cream without active component. The choice between both treatments was made at random and owners were blinded to the treatment. Packaging of the creams was identical and the labels were coded. When multiple occult or partly verrucous sarcoids were present on the same horse, all tumours were treated with the same product.

The sarcoids of both treatment groups were completely covered with cream twice daily by the owner. If cream remnants were still present, the lesion was cleaned with water and dried with a paper towel before applying more. Owners were instructed and demonstrated to beforehand how to apply the cream. Treatment continued for 6 months or until the sarcoid had disappeared completely. The experiment was stopped if sudden aggressive growth of the sarcoid would occur.

Before the start of the treatment (T0) and further at monthly intervals (T1 until T6), a close-up photograph of the sarcoid was taken with a ruler next to, but not covering the tumour. To gain more insight in the antiviral effect of acyclovir on BPV in equine sarcoids, a swab sample for BPV DNA analysis was taken at the same time as the photographs by rubbing a sterile cottontip swab soaked in sterile distilled water over the surface of the sarcoid [9]. Swabs were stored in separate containers at −20 °C until further processing. When the horses were stabled too far away from the clinic, private practitioners were responsible for obtaining the swab samples and photographs and provide them to the clinic. When this was the case, the private practitioners received clear instructions on how to do this to ensure a high sample quality.

**Fig. 1:**
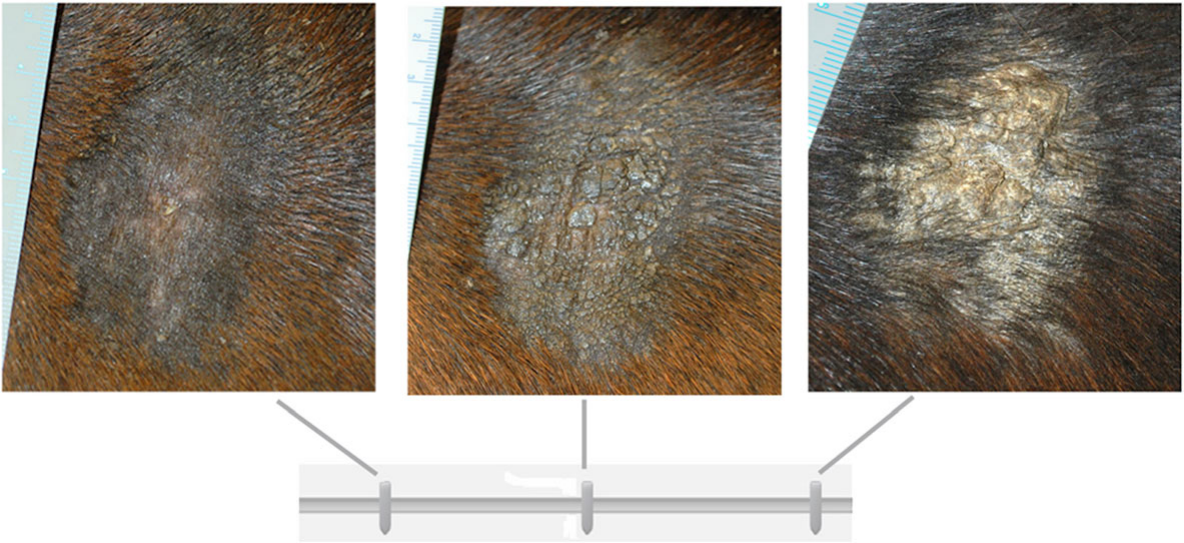
Example of how a slider bar was used to determine severity of a sarcoid on a visual analog scale (VAS), based on verrucosity

All pictures and samples were processed together at the end of the experiment. Pictures were given a random coded file name and put in random order to ensure blinded processing. On all pictures, sarcoid maximal diameter and surface area were measured twice using image measuring software (ImageJ). The mean of two measurements was used for further analysis. Severity of the sarcoid was determined by three diplomates of the European College of Veterinary Surgeons. A visual analog scale (VAS) was used ranging from no visible abnormalities of the skin (score 0) to distinct skin verrucosity (score 1000) (Fig. 1). The mean of three scores was used for further analysis.

Swabs were examined for the presence of BPV DNA. DNA was extracted using a commercial kit (DNeasy Blood & Tissue Kit, Qiagen). Swabs were first incubated for 12 h in 180 μl buffer ATL and 20 μl proteinase K at 56 °C. The samples were then vortexed and 200 μl buffer AL was added. After vortexing again, swabs were removed from the vials and discarded. Further DNA extraction was continued as described in the manual of the kit, following the tissue protocol. After DNA extraction, real-time PCR analysis was performed using general BPV primers and BPV-1 and -2 specific TaqMan probes as described by Bogaert et Al. [10]. All samples were processed in duplicate and when quantification cycle values between repeats differed by more than one, the PCR was repeated. Positive controls consisting of a mixture of known BPV-1 and BPV-2 samples and negative controls consisting of distilled water were included in each run. All samples were also tested for the presence of equine interferon beta (IFNb) DNA to confirm successful DNA extraction [11]. Samples were considered positive when either BPV-1 or BPV-2 DNA was detected. Samples were only considered negative when IFNb DNA could be detected, but no BPV DNA. When no IFNb DNA was detected, samples were marked as missing data for further analysis.

### Statistical analysis

All data analysis was performed using statistical software (SPSS 20, IBM). Statistical significance was set at *p* ≤ 0.05. To estimate the effect of treatment on the number of fully regressed sarcoids, a Fisher exact test was used. For all continuous data (sarcoid diameter, sarcoid surface area and VAS score), the effect of time and treatment on the dependent variables was determined by repeated measures ANOVAs with horse as a blocking factor. When sphericity could not be assumed, a Greenhouse-Geisser correction was applied. Additionally, a “change parameter” was calculated for continuous data from all sarcoids as the difference between the measurement at T0 and the last measurement. The effect of treatment on this “change parameter” was estimated by a generalized linear model, corrected for follow-up time and with horse as a blocking factor. For each time point, the effect of treatment on the number of samples positive for BPV DNA was tested using a binary logistic regression with horse as a blocking factor. To test the effect of time on the number of samples positive for BPV DNA, a binary logistic regression with horse as a blocking factor was used for both treatments with T0 as the reference category.

## Results

Twenty-eight horses and three ponies were included in the study. In total, 24 sarcoids on 15 individuals were treated topically with 5% acyclovir cream and 25 sarcoids on 16 individuals were treated topically with placebo cream. Multiple sarcoids were present in four horses in the acyclovir group, and in five horses in the placebo group. For both groups, median treatment time was 6 months (min: 1 month, max: 6 months). The study was stopped early because of sudden aggressive tumoural growth in one horse, which was part of the acyclovir treatment group. For three placebo treated horses and one acyclovir treated horse, the study was stopped early because of complete sarcoid regression. No side effects were observed in any of the treated horses.

Complete regression during treatment occurred in two of the acyclovir treated sarcoids (8.3%; 95% CI: 1.0% - 27.0%), while this was the case in four (16%; 95% CI: 4.5% - 36.1%) of the placebo treated sarcoid. This difference was not significant (*p* = 0.67).

**Fig. 2:**
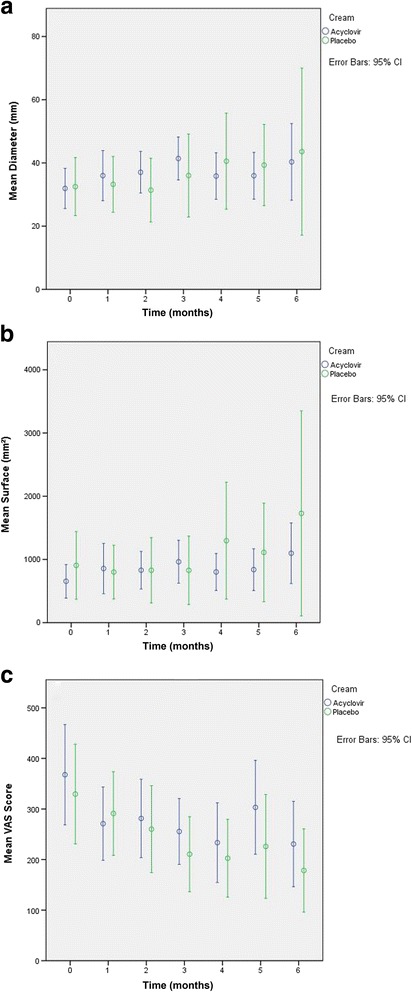
Mean measurements along with the 95% confidence interval of sarcoid diameter (**a**), sarcoid surface (**b**) and visual analog scale (VAS) score (**c**) at each time point for both treatment groups

Figure 2 shows the mean measurements along with the 95% confidence interval of sarcoid diameter (A), sarcoid surface (B) and VAS score (C) at each time point for both treatment groups. The intraclass correlation coefficient of the raters for average measures of VAS was 77.0%. There was no significant effect of treatment or time on any of these variables. The mean calculated “change parameters” are listed in Table 1 along with the *p*-values for the effect of treatment on them. A positive “change parameter” indicates a decrease in measurement between T0 and the last sample point whereas a negative change indicates an increase. The mean largest sarcoid diameter of acyclovir treated tumours increased during treatment, while it decreased for the placebo treated sarcoids (Table 1). Mean surface area increased during the treatment for both groups (Table 1). Mean VAS score decreased, indicating that sarcoids were found to be less verrucous towards the end of the treatment (Table 1). Differences in “change parameters” between both treatment groups were never significant.

**Table 1:** Mean “change parameters” and associated *p*-values for the difference between both treatment groups (SE = Standard Error; VAS = visual analog scale)

Change	Acyclovir (± SE)	Placebo (± SE)	*p*-value
Diameter (mm)	−6.10 (± 4.63)	2.02 (± 5.67)	0.52
Surface area (mm^2^)	−314.26 (± 203.44)	−60.25 (± 247.67)	0.87
VAS score	146.43 (± 53.75)	166.58 (± 39.41)	0.65

**Fig. 3:**
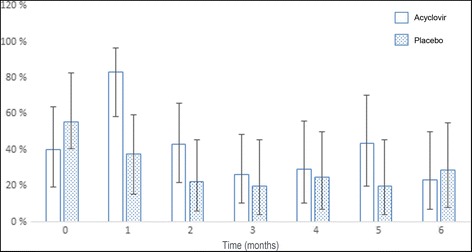
The percentage of samples positive for the presence of BPV DNA at each time point (months after start of the treatment) and corresponding 95% confidence intervals. No fill: acyclovir; dotted fill: placebo

No genomic DNA was present on the swab in 15.9% of the samples in the acyclovir treated group and in 22.8% of the samples in the placebo group. Figure 3 shows the percentage of positive PCR samples in each treatment group at each time point. Only at T1, a significantly higher percentage of samples was positive for the presence of BPV DNA in the acyclovir group compared to the placebo group (*p* = 0.005). At all other time points, there were no significant differences between groups. In the acyclovir group, the percentage of positive samples was significantly higher at T1 compared to T0 (*p* = 0.004). At all other time points, the percentage of positive samples was not significantly different from T0. In the placebo group, the percentage of positive samples was significantly lower at all time points except for T1, compared to the percentage of positive samples at T0 (T2: *p* = 0.06; T3: *p* = 0.03; T4: *p* = 0.04; T5: p = 0.04; T6: p = 0.04).

## Discussion

This is the first study on equine sarcoid treatment which is placebo controlled and double blinded. Results of this study are therefore valuable for treatment selection in practice. Because of the long study duration, the horses were cared for by their owners at home and this implicated that it was hard to check for treatment compliance. However, all owners signed a commitment to the experiment beforehand and were contacted regularly by phone to monitor the course of the study. Because the horses were stabled at home and this was in many cases too far away to allow for visits to the clinic, monthly visits were often performed by trusted private practitioners. While this implicates a possible variation in sample quality, practitioners received clear instructions on how to take pictures and swabs to maximize uniformity. A benefit of this was that the persons evaluating the pictures and analyzing the samples never saw the patients in real life, which enabled an unprejudiced evaluation.

The success rate for acyclovir treatment was lower compared to previously reported success rates [5, 8]. The sample population of the present study was smaller, which could explain the lower success rates. Moreover, acyclovir treatment was stopped after 6 months of treatment in the present study, while under normal clinical circumstances it is often continued longer when the tumour has not fully regressed yet, but a beneficial effect is observed. In both previous studies, a certain number of sarcoids have indeed been treated for over 6 months, which could have increased the success rate. While the concentration of acyclovir in the cream used in the present study was the same as in all earlier studies [5, 8], the constitution of the cream vehicle used in this and earlier studies by Haspeslagh et al. [6, 8] differed from the one used by Stadler et al. [5].

Time and treatment type did not have a significant influence on mean sarcoid dimensions or mean VAS score. Mean “change parameters” were also not significantly different between treatment groups. Nevertheless, the mean VAS score decreased for both treatment groups during the study, indicating that equine sarcoids were found less verrucous towards the end of the treatment. Perhaps the previously observed benign effect is therefore not due to the application of acyclovir, but merely to the effect of a cream that keeps the skin hydrated, preventing the formation of a thick verrucous layer. Anti-keratotic creams have analogously been used to lessen the verrucosity of equine sarcoids prior to other treatments [12]. This hypothesis can be tested by including a third “no treatment” group, which was not done here due to ethical considerations towards the owners. The presence of a keratinous layer in equine sarcoids with a high degree of verrucosity could interfere with acyclovir penetration and the presence of highly verrucous tumours could have influenced the results. Nevertheless, the mean VAS score, based on verrucosity, was not very high at T0 and decreased during treatment. VAS scores at T0 did also not differ significantly between the acyclovir and placebo group, indicating that the comparison between both groups is valid. To evaluate the effect of a thick verrucous layer on acyclovir treatment, a similar experiment could be performed comparing treatment of strictly occult versus strictly verrucous tumours.

In order to obtain a good indication of the BPV load in non-ulcerated equine sarcoids, a quantitative real-time PCR on tissue from a biopsy probably yields the most reliable results. However, this would have required the sarcoids to be biopsied prior to the study and further at each time point of evaluation, which could have influenced the outcome, as equine sarcoids often become more aggressive after being damaged [13, 14]. For this reason, swabs were obtained instead of biopsies. This also implies that histological examination to confirm the diagnosis of equine sarcoid could not be performed and that there is a chance that some lesions which regressed spontaneously were not actual equine sarcoids. Nevertheless, the clinical appearance of equine sarcoids is so typical that clinical examination by an experienced veterinarian should be sufficient to make a correct diagnosis [14, 15]. While the presence of BPV DNA can be shown in up to 100% of swabs from equine sarcoids, this is only the case in ulcerated tumours where the dermis is exposed [9]. As this was never the case in this study, the percentage of positive samples was lower and in range with the earlier reported positivity rate originating from occult sarcoids [9]. Nevertheless, in the placebo group, a clear and significant decrease in the positivity rate could be seen over time, which was not the case for acyclovir treated tumours. No plausible explanation could be given for this observation.

## Conclusions

None of the results presented in this study indicate that topical treatment of occult or partly verrucous equine sarcoids with acyclovir yields any better results compared to treatment with placebo cream.

## Abbreviations

BPV: Bovine papillomavirus
IFNb: Interferon beta
VAS: Visual analog scale
